# Face Mask Sampling for the Detection of *Mycobacterium tuberculosis* in Expelled Aerosols

**DOI:** 10.1371/journal.pone.0104921

**Published:** 2014-08-14

**Authors:** Caroline M. L. Williams, Eddy S. G. Cheah, Joanne Malkin, Hemu Patel, Jacob Otu, Kodjovi Mlaga, Jayne S. Sutherland, Martin Antonio, Nelun Perera, Gerrit Woltmann, Pranabashis Haldar, Natalie J. Garton, Michael R. Barer

**Affiliations:** 1 Department of Infection, Immunity and Inflammation, University of Leicester, Leicester, United Kingdom; 2 Department of Clinical Microbiology, University Hospitals of Leicester NHS Trust, Leicester, United Kingdom; 3 Medical Research Council Unit, Banjul, The Gambia; 4 Department of Respiratory Medicine, Glenfield Hospital, Leicester, United Kingdom; 5 National Institute of Health Research Respiratory Biomedical Research Unit, Glenfield Hospital, Leicester, United Kingdom; Fundació Institut d'Investigació en Ciències de la Salut Germans Trias i Pujol. Universitat Autònoma de Barcelona. CIBERES, Spain

## Abstract

**Background:**

Although tuberculosis is transmitted by the airborne route, direct information on the natural output of bacilli into air by source cases is very limited. We sought to address this through sampling of expelled aerosols in face masks that were subsequently analyzed for mycobacterial contamination.

**Methods:**

In series 1, 17 smear microscopy positive patients wore standard surgical face masks once or twice for periods between 10 minutes and 5 hours; mycobacterial contamination was detected using a bacteriophage assay. In series 2, 19 patients with suspected tuberculosis were studied in Leicester UK and 10 patients with at least one positive smear were studied in The Gambia. These subjects wore one FFP30 mask modified to contain a gelatin filter for one hour; this was subsequently analyzed by the Xpert MTB/RIF system.

**Results:**

In series 1, the bacteriophage assay detected live mycobacteria in 11/17 patients with wearing times between 10 and 120 minutes. Variation was seen in mask positivity and the level of contamination detected in multiple samples from the same patient. Two patients had non-tuberculous mycobacterial infections. In series 2, 13/20 patients with pulmonary tuberculosis produced positive masks and 0/9 patients with extrapulmonary or non-tuberculous diagnoses were mask positive. Overall, 65% of patients with confirmed pulmonary mycobacterial infection gave positive masks and this included 3/6 patients who received diagnostic bronchoalveolar lavages.

**Conclusion:**

Mask sampling provides a simple means of assessing mycobacterial output in non-sputum expectorant. The approach shows potential for application to the study of airborne transmission and to diagnosis.

## Introduction

Tuberculosis (TB) remains a major global health problem with 8.6 million new cases in 2012 [1]. The causal agent, *Mycobacterium tuberculosis*, is an obligate pathogen that is dependent on airborne transfer to new hosts for its long-term survival [2].A better understanding of the mechanisms involved in *M. tuberculosis* transmission offers the potential to improve public health practice and is urgent given the rising numbers of multidrug resistant (MDR) and extensively drug-resistant (XDR) strains [1].

Sputum has been the principal sample type used for microbiological diagnosis of TB [3] and enumeration of acid fast bacilli (AFB) therein has been used to assess case infectivity [4].

In early studies at the turn of the 19^th^ and 20^th^ centuries there was discussion of the relative roles of dried sputum dispersed as dust, large droplets and fine sprays together with contamination of food as key pathways for the spread of TB (reviewed in [5]). It was over fifty years later that Wells, Riley and colleagues directly demonstrated the importance of aerosolized fine droplet nuclei (<5 µm) in TB transmission [6], a view that has been further substantiated in recent airborne transmission studies [7]–[8]. However, relatively little is known about the formation of these bacteria-containing aerosols.

While it is generally assumed in clinical practice that sputum smear positive cases are the predominant sources of infection, it is self-evident that mucus-enveloped bacilli in expectorated and macroscopically visible sputum do not mediate transmission. Moreover, several recent epidemiological and experimental studies highlight the degree to which sputum positivity does not correlate with detected transmission [9]–[12].

Analytical methods that assess output of bacilli in expelled aerosols from *M. tuberculosis* infected individuals are clearly needed to enable us to investigate the mechanism of TB transmission in detail. Guinea pig infection studies in clinical facilities clearly support the possibility of droplet nuclei-based transfer over extended distances [6]–[8]. However, the feasibility of relating patient and bacterial characteristics at the time when the infectious dose is expelled to individual transmission events is very limited due to the time delay between infection and its detection in the animals. In contrast, the studies of Fennelly and colleagues have focused on output of colony-forming units (cfu) of *M. tuberculosis* in aerosols produced during brief and supervised periods of deliberate coughing [12]–[14]. These investigators have used the Cough Aerosol Sampling System (CASS) to provide important new insights into the characteristics of aerosols containing *M. tuberculosis* cfu and the relationships between individual patient CASS results and transmission. In the present context we note that, while there was a positive correlation between sputum AFB score and CASS cfu counts, the majority of AFB-positive subjects did not produce culturable aerosols and there was a tendency for patients with salivary and muco-salivary sputum samples to be aerosol cfu positive [12]. These features emphasise uncertainties in our understanding of the relationships between sputum and infectious aerosols.

While the CASS approach provides a single time point assessment of *M. tuberculosis* cfu counts in aerosols it requires significant infrastructure and carefully balanced apparatus to obtain valid samples. Moreover, the capacity of CASS to determine potentially critical assessments of TB case infectivity such as total daily output of *M. tuberculosis* and diurnal variations in output, and to achieve these analyses in settings comparable to normal daily life, are very limited.

To address these issues, we have been exploring the potential of using face masks to collect and assess expelled aerosol output from both suspected and diagnosed cases of TB. We report here our experience with mask collections from 46 individuals in different settings and mycobacterial detection achieved first by bacteriophage assay then with the GeneXpert system. At present sampling does not exclude contribution of>5 µm droplets to mask contamination. Our findings show that mask sampling can readily be used to detect *M. tuberculosis* contamination in expelled aerosols and that the approach offers potential both for the study of *M. tuberculosis* transmission and for the enhancement of microbiologic diagnosis.

## Materials and Methods

### Patients

Subjects were recruited in two series, first in Leicester (Series 1; 2007–9) then both in Leicester and in The Gambia (Series 2; 2013). Series 1 samples were collected using unmodified standard surgical masks and analyzed by bacteriophage assay. Series 2 samples were collected in rigid protective masks modified to include a filter that was subsequently processed through the Cepheid GeneXpert system.

All patients were ≥18 years and enrolled following informed consent within formation provided in their preferred language. Leicester patients were recruited though our local TB clinics, Infectious Diseases Unit and Respiratory wards at University Hospitals of Leicester NHS trust under National Research Ethics Service approval (07/Q2501/58). Gambian patients were recruited from Health Centres associated with the UK Medical Research Council (MRC) Unit in Fajara under approval SCC1343 from The Gambia Government and MRC Joint Ethics Committee. In all cases patients provided written consent following procedures specifically approved by the two ethics governance systems.

#### Series 1

Between June 2007 and March 2009, we recruited 17 patients with AFB smear positive sputum. All patients had positive confirmatory MGIT liquid cultures. Subjects were asked to wear the mask for as long as they felt comfortable with a recommended minimum of 1 hour; actual wearing times ranged from 10–300 minutes. They were instructed to talk, sneeze and cough as they wished. However, if they needed to expel sputum, they were asked to briefly remove the mask away and spit into the sputum pot provided. With the exception of 3 samples, all masks were collected after chemotherapy had begun (within 7 days).

#### Series 2

Between February and June 2013, 20 subjects were recruited in Leicester and 10 in The Gambia. Patients in Leicester were recruited on the basis of a high clinical or radiological suspicion of pulmonary TB while those in The Gambia all had at least one sputum microscopy positive for AFB prior to mask wearing. All wore filter containing masks for 1 hour. Instructions were as for the phage analyzed group except that if they did not make any vocal effort during this hour they were asked to cough once and repeat ‘Peter’ ten times. All samples were taken prior to starting TB treatment with the exception of 2 Leicester patients who were sampled on day 5. An additional sputum sample was taken from Gambian subjects during or after mask sampling and this was stained for AFB and subjected to MGIT culture.

### Analysis of Series 1 Samples – Surgical Masks and Phage Assay

**Figure 1:**
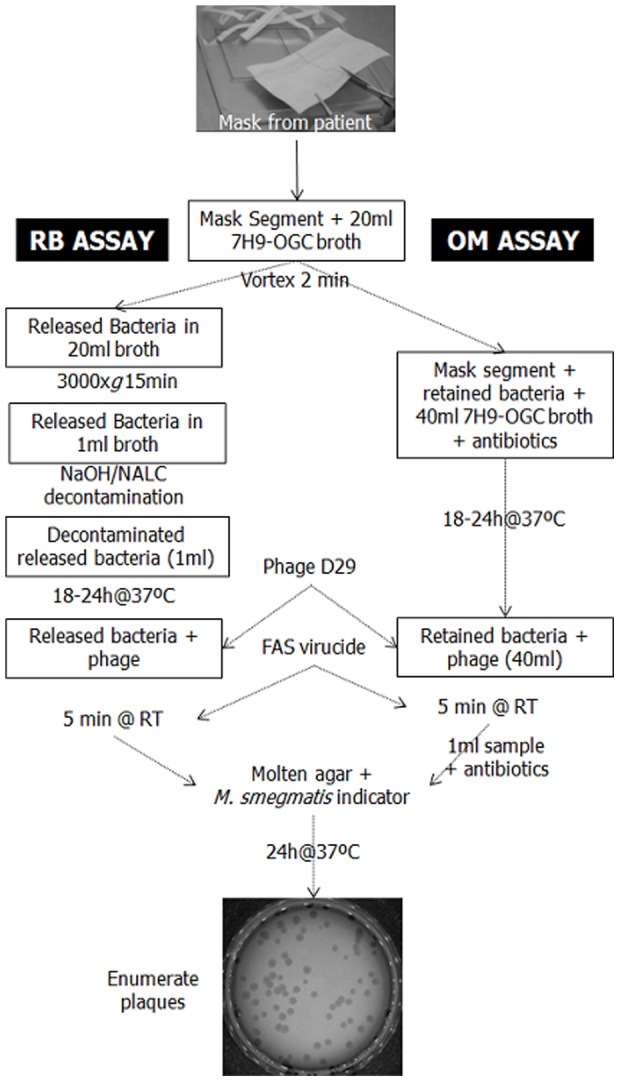
Schematic of mask processing for phage assay. See text for abbreviations.

This was a developmental and exploratory phase and two types of assay were used. For the first 9 patients mycobacteria released from the mask by vortexing were directly assayed (Released Bacteria (RB) assay). For the remaining 8 patients phage infection On Mask (OM) assay was performed on the retained bacteria. Flow charts outline the procedures for the two phage assays in Figure 1. It should be noted that the phage assay detects both *M. tuberculosis* complex and Non-Tuberculous Mycobacteria (NTM). Pleat-style masks (Kimberly-Clark surgical 48105 or procedure 47085) were used for sampling. Once worn, the mask was folded into quarters with the sampling surface facing inwards and placed into a sterile 250-ml plastic jar. Mask samples were stored at 4°C if not processed on the day of sampling. Details of the processing for the RB, OM and phage assays are given in Methods S1.

### Analysis of Series 2 samples –Modified masks and GeneXpert assay

**Figure 2:**
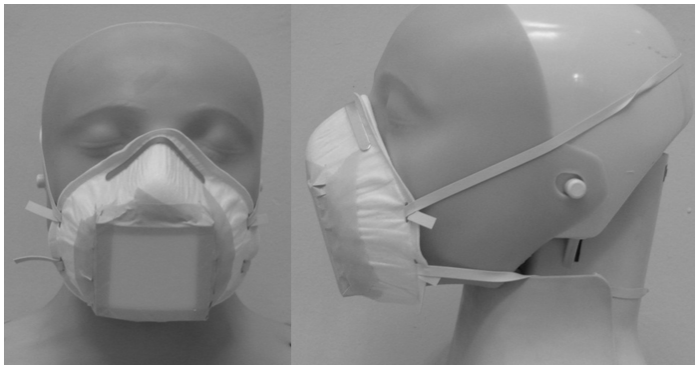
FFP30 mask with filter adapted for sampling.

FFP30 face masks (MB Filter products, Mark 30) were modified by cutting a 5.5 cm square opening from the center, into which an equivalent sized gelatin membrane (3 µm pore size, Sartorius UK) was attached in a custom-made detachable plastic holder secured with autoclave tape (Figure 2).Each mask was stored in a sterile re-sealable plastic bag which was then stored in a plastic container for no more than 5 days before being used. After sampling, the mask was placed and re-sealed into the plastic bag and plastic container and stored at -70°C until DNA extraction and PCR analysis. For analysis the filter membrane was removed from the mask, divided into 3 pieces and dissolved in 4 ml of molecular grade water (Invitrogen). This was then heated to 95°C for 10 minutes to ensure the membrane was completely dissolved. 2 ml of the dissolved filter was then transferred to a 2 ml micro-centrifuge tube and centrifuged at 10,000x*g* for 10 minutes. The supernatant was then removed and discarded and the pellet re-suspended in 1 ml of molecular grade water. This was then transferred into a 30 ml universal container to which 2 ml of the Xpert MTB/RIF sample reagent (Cepheid) was added, shaken to mix and incubated at room temperature for 15 minutes, shaking once again after 8 minutes. 2 ml of the mixture was then loaded into Xpert MTB/RIF cassettes and loaded into the GeneXpert machine and analyzed according to the manufacturer's instructions [15].

## Results

### Preliminary studies

We first explored the potential to detect mask contamination by *M. tuberculosis*-directed PCR [16] by inoculating masks with cultured bacteria. We found that extracts from the surgical masks used in series 1 were strongly inhibitory to amplification with a detection limit of ∼10^4^ colony forming units (cfu) per mask (data not shown) and the approach was abandoned and the phage assay developed. In contrast, in series 2, extracts from gelatin membranes showed little or no inhibitory activity in the same assay with a detection limit of 10^2–3^ cfu per membrane and this provided the basis for developing the GeneXpert assay.

### Series 1: Phage assays detect live bacilli on masks

Seventeen patients with at least one AFB positive sputum sample were recruited. Of these, 10 were male, 7 female, the average age was 40 years (18–70).One patient had concomitant HIV infection. All 17 had sputum culture confirmed infection, two with NTM (*M. kansasii*, patient 5; *M. avium*, patient 14) and the remaining 15 with *M. tuberculosis*.

The plaque assay results are summarized in table 1. On patients 1–9, we trialed a method in which mycobacterial cells were released from mask segments by vortexing then detected by phage assay (RB assay, see figure 1). This method gave positive mycobacterial detection on 8 occasions on samples from 6 patients. Assuming each plaque represented a single mycobacterial cell, the RB method yielded between 4 and 32 detected mycobacteria per mask (data not shown).

**Table 1:** **SERIES 1, PHAGE ASSAYS ON SURGICAL MASKS.**

Patient No.	am/pm Sample	Sampling Period (min)	Phage Assay ‡
1	am	300	-
1	pm	120	-
2	pm	120	-
3	U	10	+
3	U	U	+
4	U	60	-
4	U	60	+
5*	am	30	+
5	pm	30	-
6	am	120	+
7¶	U	50	+
7	U	25	+
8	U	40	-
9¶	U	60	+
10¶	am	30	-
10	pm	50	-
11	U	U	-
12	pm	40	+
13	am	60	+
13	pm	60	+
14†	am	60	+
14	pm	60	+
15	am	60	+
15	pm	60	+
16	am	45	+
16	pm	45	+
17	am	30	-
17	pm	30	-

ND =  not done; U =  not recorded.

*M. kansasii isolated.

† *M. avium*isolated.

‡ Patients 1–9 - RB assay; 10–17 – OM assay.

¶ Pre-chemotherapy samples.

In an effort to improve assay sensitivity we explored the value of applying phage D29 directly to contaminated masks. To our surprise, we found that this appeared to be an effective means of releasing mycobacterial cells from the mask and pfu counts well in excess of control values were readily obtained (OM assay, see figure 1). We also determined that decontamination with NaOH/NALC reduced counts by at least 3-fold (data not shown) and that assay contamination could be prevented with the combination of nystatin, oxacillin and aztreonam (NOA) recommended by Mole and colleagues [17].

The OM assay gave positive mycobacterial detection in 9 of the thirteen masks assessed and in 5 of the 8 patients. The pfu counts indicated between 6,000 and 32,000 mycobacteria per mask (data not shown).

Overall, the phage methods gave positives in 11/17 (65%) patients, all of who had clinically and microbiologically diagnosed pulmonary disease. These included two cases of NTM infection. The phage employed in our assays (D29) infects most mycobacterial species. Two masks were collected in 11 patients and discordant results (one positive one negative) were found in two cases (patients 4 and 5). Considerable variation in pfu counts was also seen when both samples were positive (6 patients; data not shown).

### Series 2: GeneXpert detects contamination of mask filter inserts in Leicester and Gambian patients

In Leicester, 20 patients with high clinical/radiological suspicion of pulmonary TB were recruited (12 males and 8 females). Average age was 39 (19–69). All were HIV negative.

One patient was excluded from analysis due to a processing error signaled by the GeneXpert instrument so that no result could be obtained. Of the remaining 19 patients, 10 (53%) were diagnosed with pulmonary TB by the consultant physician and commenced on TB treatment. Physicians were not aware of the mask result at the time of diagnosis. A further 3 patients were found to have extra-pulmonary TB, two with TB lymphadenitis and one with pleural TB, and were also commenced on TB treatment. All were notified. An alternative diagnosis was made in the remaining six patients (32%).

Of the 10 patients diagnosed with pulmonary TB, 6 were mask positive and four were mask-negative. No rifampin resistance was detected. Mask samples were negative for all patients with extra-pulmonary TB and non-TB diagnoses (Table 2).

**Table 2:** **GeneXpert ASSAY APPLIED TO FILTER INSERTS.**

Clinical Diagnosis	Patient No.	Mask GeneXpert	AFB Smear‡	Culture
Pulmonary TB (UK n = 10)	18	**+**	Sp+++	Sp +
	19	**+**	Sp SC	Sp -
	20	**+**	Sp+++	Sp +
	21	**-**	Sp SC	Sp +
	22†	+	BAL-	BAL +
	23	**-**	BAL++	BAL +
	24	**-**	BAL-	BAL +
	25	**+**	Sp-	Sp -
			BAL-	BAL +
	26	**-**	BAL-	BAL +
	27	**+**	BAL-	BAL -
Extrapulmonary* (UK n = 3)	28	**-**	LN-	LN +
	29†	**-**	PA -	PA +
	30	**-**	LN -	LN +
Non-TB (UK n = 6)	31	**-**	Sp -	Sp -
	32	**-**	Sp -	ND
	33	**-**	Sp -	Sp -
	34	**-**	Sp -	Sp -
	35	**-**	Sp -	Sp -
	36	**-**	Sp -	Sp -
Pulmonary TB (Gambia n = 10)	37	**-**	Sp -	Sp +
	38	**+**	Sp -	Sp -
	39	**+**	Sp+++	Sp +
	40	**+**	Sp++	Sp +
	41	**-**	Sp+	Sp +
	42	**+**	Sp+++	Sp +
	43	**+**	Sp+	Sp +
	44	**+**	Sp+++	Sp +
	45	**+**	Sp SC	Sp +
	46	**-**	Sp -	Sp +

BAL =  bronchoalveolar lavage; LN =  lymph node aspirate, PA =  pleural aspirate, Sp =  sputum, SC =  scanty, ND =  Not done.

‡ UK Smear result from local diagnostic service, Gambia smear result from MRC lab. All Gambian patients had a prior smear-positive from their local health clinic.

*All patients diagnosed with extrapulmonary TB were sputum smear- and culture-negative.

† Mask collected day 5 of TB treatment.

In the course of their clinical investigation, five patients were unproductive of sputum and a total of six patients had bronchoalveolar lavage aspirate samples collected to aid diagnosis. 80% (4 out of 5) of patients with a smear positive sputum result also had a mask positive result, whereas 2 out of 5 (40%) of those with an unproductive cough and a diagnosis of pulmonary TB had a mask positive result.

Sputum samples from 2 patients with GeneXpert positive mask samples failed to grow any mycobacterium in culture but a confident clinical diagnosis of TB was made. Patient 19 gave one scanty AFB positive sputum smear but remained negative by repeated culture and GeneXpert analyses applied to sputum. Patient 27 was unproductive of sputum with negative results for BAL aspirate, AFB smear and culture.

In the Gambia, 10 patients with an initial positive AFB smear were recruited. Eight patients were male, 2 female and their average age was 32 years (23–49). All patients were Black African, were of unknown HIV status, had a confident clinical diagnosis of pulmonary TB and were commenced on treatment. Seven patients had a positive mask sample. Patient 38 had 3 AFB positive smears prior to recruitment, however, the additional post-recruitment sputum was negative for both microscopy and MGIT; nonetheless, his mask was positive by GeneXpert.

In summary, of the 20 patients with clinically confirmed pulmonary TB assessed by analysis of mask filter inserts assayed by GeneXpert, 13 (65%) gave at least one positive mask. In 6 patients who had diagnostic BALs performed because suspicion of TB was high and they either did not produce sputum or this was negative, 3 gave positive filters and in one of these the BAL was negative on smear and culture. In addition, no false positives were obtained from six patients who were found to have diagnoses other than TB or from the 3 patients with extra-pulmonary TB.

Combining the phage and GeneXpert assays, mask sampling gave unambiguous evidence that infected patients expelled significant numbers of mycobacteria in aerosols in 24 out of 37 (65%) cases of pulmonary infection.

## Discussion

We have demonstrated the successful detection of *M. tuberculosis* and NTM species expectorated by patients with pulmonary disease using direct mask sampling. Masks have been shown to be contaminated with both live bacilli (phage assay) and with extractable *M. tuberculosis* DNA. Detection of the latter with the GeneXpert system potentially makes our approach available to many laboratories worldwide.

Until recently we were aware of only one previous report of mask sampling in which the RNA of respiratory viruses was detected [18]. However, our attention has been drawn to the fascinating earlier studies of L Napoleon Boston who reported in 1901 use of a face mask device in which expelled aerosols were collected on microscope slides[5]. We allude to this study further below.

In Series 1 the phage assay detected both *M. tuberculosis* and NTM with mask exposure times ranging from 10 minutes to 2 hours; at the other end of the spectrum, one patient did not produce phage detectable mask contamination after 5 hours exposure (Patient 1, table 1). Although we are confident that we detected variations in mycobacterial contamination of expelled aerosols at different time points, we only report positive and negative mycobacterial detection here since we have not specifically validated the phage assays for quantitation. However, it does appear that the OM assay was more sensitive than the RB assay and we found pfu counts indicating contamination rates of masks compatible with 10^4–5^ bacilli per hour (data not shown). Due to the small sample size and the uncertain timings of several samples we cannot draw firm conclusions concerning time of day of sampling and positive mask contamination, though morning samples have marginally more positive phage assays than afternoon samples.

In Series 2 we developed a simple rapid method in which gelatin filters incorporated into masks were exposed to expelled aerosols and subjected to GeneXpertMTB/RIF analysis. This approach gave *M. tuberculosis* positive filters in 13/20 pulmonary TB patients.

Regarding our primary purpose to establish a method for sampling *M. tuberculosis* output in expelled aerosols, mask sampling provides a simple, potentially continuous, non-invasive approach that can be used to better define the pattern of expectoration in many individuals. The phage assay demonstrated the presence of live bacilli while the molecular assay links to a widely available and WHO recommended platform. We have not sought to validate quantitative mask analyses here because the expiratory efforts of our subjects were not well-standardized and our sample size was small. However, we are confident that this can be achieved. We note mask positivity rates of 65% in both series 1 and 2 and this compares with CASS positivity rates of 25 [14], 27.7 [13]and 45% [12]. It is not surprising that the more extended sampling used here leads to higher positive rates but we emphasize that the mask approach could allow the natural pattern of output to be studied. In contrast, the CASS approach probes the capacity of an individual to produce an infectious aerosol only at a single time-point.

By sampling expelled aerosols, masks incorporate both aerosol and large (>5 µm) droplets. Combining daily mask output with sputum output should allow estimation of the total number of bacilli expectorated per day. Given the recent recognition of multiple *M. tuberculosis* phenotypes in sputum and indications that many bacilli are not replicating [19], [20], combined mask and sputum enumeration could facilitate insight into the pattern of intrapulmonary replication necessary to achieve the observed output.

It is widely accepted that production of *M. tuberculosis* contaminated droplet nuclei is the principal mode of transfer to the lungs of a new host. Whether or not the mixed droplet and aerosol assessment achieved by mask sampling will undermine the capacity to relate results to transmission is yet to be determined. However, droplet mediated transmission of TB to intimate contacts cannot be excluded. This mode of transmission contributes to a number of upper and lower respiratory tract infections [21]–[23] and we are not aware of data ruling this out in TB. While it seems likely that fine aerosols are responsible for most transmission, the specific relationship between large and fine aerosols of *M. tuberculosis*, to our knowledge, has not been studied. Since humans produce a wide range of aerosol particle sizes [24], the mask sampling approach may offer a useful tool to study the relationship between expelled aerosols of all sizes and infectivity. The approach is also amenable to refinement in the filter capture system such that some degree of droplet/aerosol discrimination is possible.

In a diagnostic context, the numbers of patients and controls studied here are insufficient to make formal estimates of sensitivity and specificity. However, using clinical diagnosis of pulmonary TB for comparison, we have no evidence of false positives in either series. With the design shown in figure 2, care would be needed to exclude exogenous contamination in high burden settings particularly when masks are worn in the home environment. This could be achieved by covering the external surface of the filter and by strict instructions regarding storage. Regarding false negativity, seven of 20 mask samples in series 2 were negative in patients whose diagnosis was confirmed by culture. To improve the sensitivity of mask sampling for diagnosis there is clear scope to increase sampling times and apply a more sensitive molecular assay [25], [26].

We obtained positive masks from 3 of 6 patients in whom BAL sampling was performed. This raises the possibility that mask sampling might obviate the need for this costly and invasive procedure in some cases. In resource poor settings where BAL sampling is not performed, mask sampling may offer an alternative diagnostic tool for smear negative or non-productive patients.

### Limitations of the study

In addition to the relatively small sample size, interpretation of our results is limited by the developmental nature of the work leading to evolving methodologies and differing inclusion criteria. There is much scope with this approach to determine the optimum duration and time of day for sampling as well as defining or monitoring the expiratory effort associated with each sample. We have also noted that a formal study designed to determine diagnostic value would need to take precautions regarding potential exogenous contamination. Further developments could focus on optimizing the sensitivity and quantitative aspects of the molecular assay.

Finally, relating our results to the studies of L Napoleon Boston [5], he reported that 38 of the 50 pulmonary patients he studied yielded slides positive for tubercle bacilli detected by carbol-fuchsin staining with exposure times of 1–1.5 hours; the study was organized “with the object being not to collect on the slide the spray produced by vigorous coughing”. He comments that “In fully one-third of positive cases the bacilli were very numerous”. Regarding the negative slides, he notes that 7 of these related to patients with paucibacillary contemporaneous sputum samples. Given that more than a century separates our studies and the very likely advanced pathology in pre-chemotherapy patients, the correspondence in positivity rates of 76 and 65% respectively for Boston and the present study is intriguing. Moreover, the low sensitivity of microscopy for detecting bacilli makes it all the more remarkable that such a high frequency of positives was obtained. Indeed, many of his patients seem to have expelled aerosols contaminated at the highest levels we have estimated.In summary, mask sampling is a simple and amenable approach to monitoring respiratory output of infectious particles. Samples can be subjected to biological or molecular assays and the approach has potential to serve both research and diagnostic applications.

## Supporting Information

Methods S1 Bacteriophage detection method.
(DOCX)
