# Supplementary material for: Face Mask Sampling for the Detection of Mycobacterium tuberculosis in Expelled Aerosols
Source: PLoS One. 2014 Aug 14;9(8):e104921. doi: 10.1371/journal.pone.0104921 (PMC4133242; doi:10.1371/journal.pone.0104921)
Supplement: Methods S1 — Bacteriophage detection method. (DOCX) [file pone.0104921.s001.docx]

*RB Assay (with NaOH decontamination):* On removal, the mask was cut into 2 equal segments. Each segment was transferred into 20ml Middlebrook 7H9-OGC broth (7H9 broth + 10% v/v OADC and 1mM CaCl_2_) in sterile 50ml Falcon tubes and vortexed for 2 minutes. After vortexing, the mask segment was removed and the eluate centrifuged at 3000x*g* for 15 minutes and the supernatant removed leaving approximately 1ml residue into which the pellet was re-suspended. To this 1ml 2% w/v NaOH-NALC was added and the suspension incubated at room temperature for 15 minutes then vortexed for 30 seconds. The sample was then neutralized with 20ml of 67mM phosphate buffer and the tube contents centrifuged at 3000x*g* for 15minutes. The resulting pellet was washed once in 2ml of Middlebrook 7H9-OGC, centrifuged again and resuspended in 1ml of 7H9-OGC, incubated at 37°C for 18-24 hours then subjected to phage assay.

*OM Assay-(no decontamination):* Initial processing was identical to the RB procedure then, following vortexing, removed mask segments were each added to 40ml 7H9-OGC broth containing antibiotic supplement (nystatin 10 µg/ml, oxacillin 2µg/ml, aztreonam 30µg/ml, Biotec) and incubated overnight at 37°C prior to phage assay.

*Phage assay:* The assay was based on that described by McNerny and colleagues with minor modifications [1] for the RB and OM procedures. In both cases mycobacteriophage D29 was added directly to the overnight incubated preparations (released bacteria in 1 ml medium for the RB assay and mask segment with attached bacteria in 40 ml medium for the OM assay) to a final concentration of 3 x 10^7^ PFU/ml and the reaction incubated at 37°C for 1 hour. Ferrous ammonium sulfate (virucide) was then added to a final concentration of 8.3 mM and the reaction incubated at room temperature for 5 min. For the RB assay, the virucide treated preparation was then mixed with 9 ml of 7H9-OGC molten agar (broth +1.5% w/v agar powder) and 1ml of indicator *Mycobacterium smegmatis* mc^2^155 culture (shaken 24 hour culture in ADC supplemented 7H9 from a starting OD_580_ = 0.05). For the OM assay, the mask segment was removed and discarded and 1ml of the remaining tube contents was plated out as above but the agar also contained nystatin 37.5 µg/ml, oxacillin 512 µg/ml, and aztreonam 30 µg/ml. Plates were left to set then incubated at 37°C overnight and resulting plaques enumerated. Plaque forming unit (pfu) counts were compared between patient worn and control unexposed masks. For the RB assay, control pfu counts were uniformly negative while, for the OM assay a few background pfu were seen but the count on the exposed mask exceeded that seen on the control by at least 4-fold. (Note: For patients 1 and 2 the BIOTEC FASTPlaqueTB^TM^ (FPTB) Assay kit was used in the RB assay.)

**References**

1. McNerney R, Kambashi BS, Kinkese J, Tembwe R, Godfrey-Faussett P. (2004) Development of a bacteriophage phage replication assay for diagnosis of pulmonary tuberculosis. J Clin Microbiol 42: 2115-2120.
